# The epidemiology of *Taenia* spp. infection and *Taenia solium* cysticerci exposure in humans in the Central Highlands of Vietnam

**DOI:** 10.1186/s12879-018-3434-9

**Published:** 2018-10-22

**Authors:** Dinh Ng-Nguyen, Mark Anthony Stevenson, Kathleen Breen, Trong Van Phan, Van-Anh Thi Nguyen, Tinh Van Vo, Rebecca Justine Traub

**Affiliations:** 1 Faculty of Veterinary and Agricultural Sciences, University of Melbourne, Parkville, VIC 3052 Australia; 2 Faculty of Animal Sciences and Veterinary Medicine, Tay Nguyen University, Dak Lak, Vietnam; 3 Department of Livestock, Montana Veterinary Diagnostic Lab, Bozeman, MT USA; 4 Faculty of Medicine and Pharmacy, Tay Nguyen University, Dak Lak, Vietnam; 5 Department of Physiology, Pathology and Immunology, Pham Ngoc Thach University of Medicine, Ho Chi Minh, Vietnam

**Keywords:** Epidemiology, Taeniasis, Cysticercosis, Risk factors, Vietnam

## Abstract

**Background:**

Vietnam is endemic for taeniasis and *T. solium* cysticercosis. Despite this, information on the epidemiological characteristics of the diseases in the Central Highlands of Vietnam are poorly described. The aims of this study were to determine the epidemiological characteristics of taeniasis (*Taenia* spp.) and *T. solium* cysticerci exposure in humans in Dak Lak province in the Central Highlands, Vietnam.

**Methods:**

This cross-sectional study was carried out in six villages in three districts of Dak Lak. A total of 190 households were visited. From each household, between one and five individuals were asked to donate a single faecal and blood sample and respond to a questionnaire. Serum samples were subjected to lentil lectin purified glycoprotein enzyme-linked immunoelectrotransfer blot assay to detect antibodies against *T. solium* cysticerci. Multiplex real-time PCR was used to detect *Taenia* spp. infection in faecal samples. A fixed-effects logistic regression model was developed to identify factors associated with the probability of *Taenia* spp. infection or *T. solium* cysticerci exposure risk. The contribution of each of identified factor was quantified using population attributable fractions.

**Results:**

The prevalence of seroexposure to *T. solium* in Dak Lak was 5% (95% CI 3% to 8%). Consumption of raw vegetables, sourcing drinking water from lakes, streams or ponds and the practice of outdoor defaecation were identified as primary risk factors for the prevalence of *T. solium* cysticerci exposure, while consuming undercooked pork and beef, pork tongue and observing *Taenia* proglottids in stool were associated with *Taenia* spp. infection. Consumption of raw vegetables attributed to 74% of *T. solium* cysticerci exposure-positive cases and consumption of undercooked beef attributed to 77% of taeniasis cases in these communities.

**Conclusions:**

The prevalence of *T. solium* seroexposure in Dak Lak is consistent with those reported in other regions of Vietnam. The identified risk factors associated with the prevalence of *T. solium* seroexposure and taeniasis infection in Dak Lak are modifiable and thus advocate for targeted community intervention programs to mitigating these risks.

**Electronic supplementary material:**

The online version of this article (10.1186/s12879-018-3434-9) contains supplementary material, which is available to authorized users.

## Background

*Taenia solium* cysticercosis and human taeniasis are considered neglected tropical diseases by the World Health Organization [1]. When accidentally ingested either directly, or indirectly via contaminated food and water, eggs of *T. solium* hatch and lodge in the skeletal muscle, under the skin and/or within the central nervous system causing muscular-, subcutaneous- and/or neuro-cysticercosis. Neurocysticercosis is a common cause of epilepsy, syncope, paralysis and chronic headache [2, 3]. Globally, the disease has been estimated to cause approximately 2.8 million DALYs (disability-adjusted life-years) lost and between 2010 and 2015 approximately 300,000 individuals were estimated to be infected globally, resulting in over 28,100 deaths [4]. Consumption of raw or undercooked meat and/or visceral organs containing cysticerci of *T. solium*, *Taenia saginata* or *Taenia asiatica* cause taeniasis. Taeniasis reduces the quality of life, is responsible for diagnostic and treatment costs in infected individuals and indirectly contributes to poverty due to losses in livestock production arising from organ condemnation and loss of market access [5].

Vietnam is considered endemic for taeniasis and *T. solium* cysticercosis [6, 7]. Previous research focusing on these diseases in Vietnam has mostly been carried out in the north of the country. A review of the literature shows that common risk factors for taeniasis and cysticercosis include consumption of raw or undercooked meat and vegetables, lack of functioning latrines, husbandry practices allowing pigs to free-roam and routine use of human waste for fertilizing and irrigating crops [8–12]. Information on the prevalence of and risk factors for taeniasis and *T. solium* cysticercosis in the central and southern areas of Vietnam is scarce and outdated [12]. In Dak Lak province, there is no information available on the infection status of *T. saginata* in livestock, however our research data on the seroprevalence of cysticercosis in pigs was 0.95% [13]. A previous study carried out by our group using a newly developed multiplex real-time PCR found the prevalence of *Taenia solium, T. saginata* and *T. asiatica* taeniasis to be 1.2%, 5.8% and 1.5%, respectively in Dak Lak province [14]. In this study, our aims were to: i) estimate the seroprevalence of *T. solium* cysticerci exposure using the lentil-lectin purified glycoprotein enzyme-linked immunoelectrotransfer blot (LLGP-EITB) assay; ii) identify and quantify risk factors for human *T. solium* cysticerci exposure and taeniasis (*Taenia* spp. infection), that inform optimal risk mitigation measures in studied communities in Dak Lak province, Vietnam.

## Methods

### Studying site and sampling

The study was deployed in the districts of Buon Don, Krong Nang and M’Drak in the province of Dak Lak in the Central Highlands of Vietnam between May and October 2015. M’Drak is located in the east of Dak Lak province with an average altitude of 400 m to 500 m and has a tropical monsoon climate typical of the Vietnam’s Central Coast. Krong Nang is situated in the north of the province at an altitude of 800 m. Buon Don, situated to the west of the province with an average elevation of 330 m and has a hot and dry climate. The standard of living in this area of Vietnam is generally poor. The literacy rate among local residents aged between 15 and 60 in rural areas was low [15]. Open defaecation using outdoor pit latrines is common practice and livestock access to these latrine areas is usually unrestricted. The practice of non-confinement of pigs and cattle is common with slaughter activities often carried out in backyards [12]. The sample size was calculated based on the previous study of Van De at al. [31]. The seroprevalence of *T. solium* cysticerci exposure was assumed to be 7% and there was 95% certainty that this estimate was within 5% of the true population value (i.e. the prevalence ranged from 2% to 12%). Ignoring the tendency for *T. solium* cysticerci exposure to cluster within households we estimated that a total of 100 samples were required. We then assumed the average number of individuals eligible for the study per household was three and the between household (cluster) variance in seroprevalence was around 1.14 times greater than the within household (cluster) variance, returning an intra-class correlation coefficient of 0.07 [16]. Our revised sample size, accounting for the tendency of cluster within households, was 114 for each district and 342 for the whole province

A sampling frame listing the name of all villages in Dak Lak province was obtained from the Sub-Department of Animal Health office within the Ministry of Agriculture. Villages eligible for sampling comprised those with more than 1000 pigs, as recorded by the Sub-Department of Animal Health. All eligible villages within each district were assigned a number and two numbers chosen at random to select villages from each district for inclusion in the study. A list of householder names within each selected village was obtained from each village head person, and household names were assigned a numeric code. The sheet of paper of numeric household codes for each village were cut into pieces and placed face-down on a table. The village head person was asked to select 50 households at random. Each village has a number of households between 200 and 300. All selected households were visited several days before the proposed sampling date to obtain consent from participants to take part in the study. From each household between one and five individuals were requested to take part in the study. Individuals eligible for inclusion in the study were healthy, not pregnant and over seven years of age. Individuals requested to take part in the study signed a consent form. Those that were under 18 years of age were required to provide written consent as well as written consent from either their parents or legal guardians. At the time of consent, each study participant was given a labeled stool container, with instructions that the container would be collected on the date of sampling, several days later. Five mL of venous blood was collected into plain clotting tubes from consenting study participants by local medical staff. The blood was kept at ambient temperature for clotting and then centrifuged at 3200×g for 5 min to collect serum. The serum was stored at − 20 °C until use.

### Household questionnaire

Each study participant was requested to answer a prepared questionnaire (Additional File 1). The questionnaire comprised of two sections. The first section collected information at the household level and was completed by the nominated head of the household. Questions included in this section solicited details on housing type, details of the type of latrines in use (if any), the presence and type of backyard pigsties, the source(s) of drinking water and activities associated with cultivation, irrigation and fertilization of home or farm grown vegetables. The second part of the questionnaire solicited details at the individual study participant level including their age, gender, the highest level of education achieved, dietary practices, and hygiene. All questionnaires were conducted in local Vietnamese phraseology and the validity of the questionnaire was pre-tested on thirty individuals in another community in Dak Lak province before application to the field survey. In addition, interviewers were trained before administering the questionnaires.

### Laboratory procedures

#### Detection of antibodies against *T. solium* cysticerci

Serum samples were subjected to LLGP-EITB assay to detect antibodies against *T. solium* cysticerci. This assay has a diagnostic sensitivity of 98% and a diagnostic specificity of 100%. The assay was performed as previously described by Tsang et al. (1989) [17] and Noh et al. (2014) [18].

#### Detection of *Taenia* spp. infection

A total of 342 faecal samples were subject to a multiplex real-time PCR (T3qPCR) to detect all three species of *Taenia*, notably, *T. solium*, *Taenia saginata* and *Taenia asiatica*. The assay has been reported to have a diagnostic sensitivity of 94% and a diagnostic specificity of 98%. The positive results of T3qPCR were confirmed using either a conventional multiplex PCR targeting *T. solium*, *T. saginata* and *T. asiatica* [19] or a singleplex PCR for *Taenia* sp. following DNA sequencing. The methods and results have been described and published elsewhere [14].

### Statistical methods

Risk factors for *T. solium* cysticerci exposure and taeniasis (*Taenia* spp. infection) in humans in the communities of Dak Lak province were identified using logistic regression. The outcome of interest for this study was a binary response variable, Y_i_, taking the value 1 if an individual was *T. solium* cysticerci exposure-positive using the LLGP-EITB or in the case of taeniasis, was positive for *Taenia* spp. infection by T3qPCR, and 0 otherwise.

This study did not use a simple random sampling design to avoid erroneous point estimates of the strength of the association between hypothesized risk factors and the outcome of interest and their standard errors. The data were analyzed in a manner that respected the three-stage cluster design (villages within districts formed the primary sampling units, households within villages formed the secondary sampling units and individuals within households formed the tertiary sampling units). Sampling weights *W*_*I*_, provided an estimate of the inverse probability of an individual being selected for study (Lumley 2010) [20] and were quantified for each study participant as follows:

1 $$ {w}_1=\frac{V}{v}x\frac{H}{h}x\frac{M}{m} $$

In Eq. 1, *V* and *v* represent the total number of villages in each district and the number of sampled villages in each district, respectively; *H* and *h* represent the total number of households in each sampled village and the number of sampled households in each village, respectively; and *M* and *m* represent the total number of individuals in each sampled household, and the number of sampled individuals in each household, respectively.

Unconditional associations between each of the hypothesized risk factors from the questionnaire and the outcome of interest were computed using the odds ratio. Hypothesized risk factors (explanatory variables) with unconditional associations that were significant at *P* <  0.20 level (2-sided) were selected for multivariable modeling.

A fixed-effects logistic regression model was developed where the probability of an individual being *Taenia*- or *T. solium* cysticerci exposure-positive was parameterized as a function of the explanatory variables with unconditional associations significant at P <  0.20, as described above. Explanatory variables that were not statistically significant were removed from the model one at a time, beginning with the least significant, until the estimated regression coefficients for the explanatory variables retained were significant at an alpha level of less than 0.05. Explanatory variables that were excluded at the initial screening stage were tested for inclusion in the final model and were retained if their inclusion changed any of the estimated regression coefficients by more than 20%. Biologically plausible two-way interactions were tested and none were significant at an alpha level of 0.05.

To account for the hierarchical structure of the data, that is, villages within districts and households within villages and study participants within households, we extended the model to include village- and household-level random effect terms using the lme4 package [21] in R version 3.4.3 [22]. This extension to the model was informative because it provided the opportunity to distinguish the influence of unobserved explanatory variables operating at the village, household and individual level on *Taenia-* and *T. solium* cysticerci exposure-positive risk. In our multilevel model the variance of the random effect terms at the village and household level were negligible, and the point estimates of the regression coefficients and standard errors for the explanatory variables were identical to the fixed-effects model. For parsimony, we report the results of the fixed-effects regression model adjusted for the three-stage sampling design using the survey package [23] in R.

The contribution of each of the explanatory variables in the final model on *Taenia-* and *T. solium* cysticerci exposure-positive risk was quantified using the population attributable fraction. The population attributable fraction for a given explanatory variable is the proportional reduction in outcome event incidence expected by either eliminating exposure to the variable or completely preventing the effects of exposure, assuming the explanatory variable is causative and assuming there has been no bias and sampling error in the study population [24]. Using the method of Toschke et al. (2007) [25], adjusted population attributable fractions were calculated for each of the explanatory variables that were significant in the mixed-effects logistic regression model.

## Results

### General description of study population and data structure

Table 1 presents the structure of the data. In total 342 individuals, from 190 households in six villages in three districts of Dak Lak province consented to take part in the study. A household had, on average, two individuals that consented to take part (minimum 1; maximum 5). In total, only 25% (95% confidence interval (CI) 19% to 32%, 47 of 190) of households possessed a latrine and 25% (95% CI 19% to 32%, 48 of 190) of households sourced their water for daily activities, including drinking, from lakes, streams or ponds. Untreated drinking water was consumed by 60% (95% CI 53% to 67%, 115 of 190) of households (Table 2). The literacy rate among participants was 56% (95% CI 51% to 62%, 193 of 342). Most participants (87%, 95% CI 80% to 88%) listed farming as their main occupational activity (Table 3). The percentage of study participants that did not wash their hands after defaecation or before eating was 35% (95% CI 31% to 41%, 121 of 339) and 44% (95% CI 39% to 50%, 150 of 339), respectively.

**Table 1:** Structure of the data from 342 study participants from six villages in M’Drak, Buon Don and Krong Nang districts

Level	Number	Number at the next highest level	
		Mean	Range
Districts	3	–	–
Villages	6	2	2
Households	190	31	18 to 40
Humans^a^	342	2	1 to 5

^a^A total of 342 individuals participated in this study. The mean of individuals per household was 2 (range 1 to 5)

**Table 2:** General description of household data

Characteristic	Frequency	Percentage (95% CI)
Number of households	190	
District		
M’Drak	58	30 (24 to 38)
Buon Don	66	35 (28 to 42)
Krong Nang	66	35 (28 to 42)
Owning pigs		
Yes	37	19 (14 to 26)
No	153	81 (74 to 86)
Presence of latrine		
Yes	47	25 (19 to 32)
No	143	75 (68 to 81)
Source of drinking water		
Pipe/well/rain water	142	74 (66 to 79)
Lake/stream/pond	48	26 (19 to 32)
Water treatment		
Yes	75	39 (32 to 47)
No	115	60 (53 to 67)
Are vegetables fertilized?		
Yes	81	43 (35 to 50)
No	109	57 (50 to 64)

**Table 3:** Demographic data for individual study participants

Characteristic	Frequency	Percentage (95% CI)
Total number of participants	342	
Age (years)		
< 25	63	18 (14 to 23)
25–60	223	65 (60 to 70)
> 60	56	16 (13 to 21)
Sex		
Male	104	30 (26 to 36)
Female	238	70 (64 to 74)
Highest level of education		
None	149	44 (38 to 49)
Primary/secondary	184	54 (48 to 59)
Tertiary	9	2.6 (1.3 to 5.1)
Primary activity		
Farming	298	87 (83 to 90)
Livestock	14	4.1 (2.3 to 6.9)
Civil service	5	1.5 (0.5 to 3.6)
Other	25	7.3 (4.9 to 11)
Ethnicity		
Kinh	40	11 (8.6 to 15)
Ede	302	88 (84 to 91)

Total of 342 serum samples collected from study participants, three were excluded due to hemolysis resulting in 339 samples included for further analysis. Of 339 serum samples, antibodies against *T. solium* cysticerci were identified in 17 individuals (5%, 95% CI 3% to 8%)*.* Among 342 faecal samples, 23 (6.7%, 95% CI 4.4% to 10%) were positive for *Taenia* spp. Single infection of *T. solium* and *T. saginata* were identified in three and 14 samples, respectively; mixed infections of *T. solium* and *T. saginata* occurred in one samples, and of *T. saginata* and *T. asiatica* in five samples [14].

### Risk factors for *T. solium* cysticerci exposure

Of the data recorded using the questionnaire, we identified three factors associated with an individual’s likelihood of being exposure-positive to *T. solium* cysticerci based on the results of the LLGP-EITB assay. These were, the frequent consumption of raw vegetables, sourcing drinking water from lakes, streams or ponds and outdoor defaecation.

Estimated regression coefficients for consumption of raw vegetables, the source of drinking water and routine location of defaecation are provided in Table 4. After adjusting for other explanatory variables in the model, the odds of an individual who often consumed raw vegetables being *T. solium* cysticerci exposure-positive was 9.98 (95% CI 2.89 to 34.4) times that of an individual who consumed raw vegetables rarely. The odds ratio for those who routinely defaecated outdoors was similar, at 11.1 (95% CI 2.97 to 42.0). The adjusted population attributable fraction for the consumption of raw vegetables was 74% and thus, reducing the prevalence of raw vegetable consumption in the population is expected to reduce the exposure to *T. solium* cysticerci by a factor of 74%. Similarly, the population attributable fraction estimate for routine location of defaecation was 60%.

**Table 4:** Risk factors associated with *T. solium* cysticerci exposure identified by a LLGP-EITB assay

Explanatory variable	Number positive	Number of individuals	Regression coefficient (SE)	*P* value	Adjusted OR (95% CI)
Intercept	17	339	−6.4694 (1.0615)	< 0.01	–
Raw vegetable consumption				< 0.01	
Occasionally	4	226	Reference		1.00
Often	13	113	2.3006 (0.6318)		9.98 (2.89 to 34.4)^a^
Area of defaecation^b^				< 0.01	
Close latrine	5	228	Reference		1.00
Outdoor	12	110	2.4125 (0.6763)		11.1 (2.97 to 42.0)
Source of drinking water				< 0.05	
Other^c^	9	253	Reference		1.00
Stream/Lake/Pond	8	86	1.3702 (0.5684)		3.94 (1.29 to 11.9)

^a^Interpretation: After adjusting for other explanatory variables in the model, the odds of an individual who consumed raw vegetables often being *T. solium* cysticerci exposure was 9.98 (95% CI 2.89 to 34.4) times that of an individual who consumed rare vegetables rarely

^b^A single individual with a missing value for area of defaecation excluded

^c^Drinking water sourced from wells, pipes, rainy and packed water

The odds of *T. solium* cysticerci exposure for those that routinely sourced their drinking water from streams, ponds or lakes was 3.94 (95% CI 1.29 to 11.9) times higher than the odds of *T. solium* cysticerci exposure for those that sourced their water from wells.

### Risk factors for *Taenia* spp. infection

We identified five factors associated with an individual’s likelihood of infection with *Taenia* spp. gender, the frequent consumption of pork tongue, the frequent consumption of undercooked pork, frequent consumption of undercooked beef, and observation of *Taenia* proglottids shedding from either themselves, their relatives or neighbors (Table 5).

**Table 5:** Logistic regression model showing the effect of sex, dietary and observation of proglottids on the odds of a taeniasis case

Explanatory variable	Number positive	Number of individuals	Regression coefficient (SE)	*P* value	Adjusted OR (95% CI)
Intercept	23	342	−6.6069 (1.0365)	< 0.01	–
Sex:				< 0.05	
Female	8	238	Reference		1.00
Male	15	104	1.0183 (0.5209)		2.77 (1.00 to 7.68) ^a^
Undercooked pork consumed:				< 0.05	
No	9	241	Reference		1.00
Yes	14	101	1.3023 (0.5695)		3.68 (1.20 to 11.2)
Pork tongue consumed				< 0.01	
Rare	15	305	Reference		1.00
Often	8	37	1.5309 (0.5778)		4.62 (1.49 to 14.3)
Undercooked beef consumed				< 0.01	
No	2	154	Reference		1.00
Yes	21	188	1.935 (0.8070)		6.92 (1.42 to 33.7)
Proglottids observed				< 0.01	
No	12	302	Reference		1.00
Yes	11	40	2.592 (0.5842)		13.36 (4.25 to 41.9)

^a^Interpretation: After adjusting for other explanatory variables in the model, the odds of an individual who is male being taeniasis infection was 2.77 (95% CI 1.00 to 7.68) times that of an individual who is female

After adjusting for other explanatory variables in the model, the odds of *Taenia* spp. infection for an individual who frequently consumed pork tongue was 4.62 (95% CI 1.49 to 14.3) times the odds for an individual who consumed pork tongue only rarely. Males had higher odds of being *Taenia* spp. positive compared with females (OR 2.77, 95% CI 1.00 to 7.68).

Among the modifiable risk factors for *Taenia* spp. infection identified in this study, the adjusted population attributable fraction for frequent consumption of undercooked beef was 77%. The population attributable fractions for frequent consumption of undercooked pork and pork tongue were 24% and 26%, respectively. Assuming undercooked beef consumption is a component cause of taeniasis risk, reducing the prevalence of this practice in the population is expected to reduce the overall prevalence of taeniasis by a factor of 77%.

## Discussion

The seroprevalence of exposure to *T. solium* in rural communities in Dak Lak in the Central Highlands was 5% (95% CI 3% to 8%). This prevalence is relatively similar to other regions across Vietnam. Phan et al. (2001) [11] reported a 4% seroprevalence of cysticercosis across 22 provinces in the south of Vietnam. For studies in the north and northwest, where *T. solium* is reported to be endemic, the seroprevalence ranged from 2 to 6% [26–29]. In this study, various risk factors were evaluated to determine their association with seroexposure to *T. solium* using the LLGP-EITB assay. Of these, raw vegetable consumption, the source of drinking water and routine location of defaecation were significantly associated with an increase in *T. solium* cysticerci exposure (Table 4). It is known that risk factors for transmission and circulation of *T. solium* cysticercosis are numerous and may vary in different settings. The identified risk factors in Dak Lak were consistent with that of study carried out in the north and south of Vietnam [11, 30]. In contrast to studies in Vietnam’s north, the utilisation of night-soil was not associated with the increase in *T. solium* exposure observed in these study communities [12, 31]. Similar to the studies in western Sichuan (China) [32], Mbeya (Tanzania) [26] and northern India [33], routine open defaecation, utilisation of unsafe water sources and consumption of raw vegetables significantly increased the risk of cysticercosis in Dak Lak. However, Cherian et al. (2014) [34] reported that these factors were not contributive in individuals who had epilepsy in Kerala, India. Research on the epidemiological characteristics of human *T. solium* cysticercosis in Nigeria [35], Tanzania [36], and Guatemala [37] found that gender and age [38] were associated with cysticercosis seropositivity, in which the female gender and increased age were significantly linked to the prevalence of *T. solium*. In our study, age and gender did not contribute to seroexposure of *T. solium* cysticerci.

Each of the identified risk factors in this study are eminently modifiable and if effective interventions are deployed, we predict that there will be marked reduction (in the order of 60% to 74%) in the prevalence of exposure to *T. solium* cysticerci in this area of Vietnam. Dak Lak province has a population of approximately 1.8 million [39]; the survey adjusted apparent prevalence of exposure to *T. solium* cysticerci, as estimated in this study, at 5.01% equates to approximately 90,000 exposure-positive individuals. With a population attributable fraction for routine location of defaecation at 60%, and assuming an intervention program to educate inhabitants to practice defaecation indoors, we estimate that the number of exposure-positive individuals will be reduced to approximately 36,000 individuals if the program is 100% successful. Even with 50% efficacy of the intervention program, we estimate the number of exposure-positive individuals would be in the order of 63,000, a substantial reduction from the current prevalence estimate of 90,000. The expected savings to the public health sector (in terms of avoided diagnostics and treatments) is likely to be substantial. If both location of defaecation and raw vegetable consumption practices are modified concurrently, the expected population attributable fraction is 89%. Outdoor defaecation results in the contamination of the environment including water and vegetables; thus, to reduce or mitigate the burden of *T. solium* cysticerci exposure of inhabitants in the communities of Dak Lak province, this factor should be given the highest priority of implementation as an intervention program.

*Taenia* spp. infection risk in in this study was associated with gender, the consumption of pork tongue, consumption of undercooked pork and beef, and observation of *Taenia* proglottids shedding from either themselves, their relatives or neighbors (Table 5). Among these factors, the consumption of pork tongue, and consumption of undercooked pork and beef are modifiable. Consumption of undercooked beef had the highest population attributable fraction of 77% compared to other factors which ranged from 24 to 26%. Thus, an intervention program aimed at discouraging practices of consuming undercooked beef could potentially result in a 77% reduction in the number of cases of taeniasis in the region. The strong association of undercooked beef consumption with *Taenia* positivity could explain the dominant prevalence of *T. saginata* in this study population at 5.8% compared to 1.2% with *T. solium* and 1.5% with *T. asiatica* [14], because beef containing *T. saginata* cysticerci is the cause of *T. saginata* taeniasis in humans.

Males had a higher likelihood of taeniasis compared to females. In rural communities of Dak Lak, males commonly consume undercooked beef, pork, or pork visceral organs while drinking alcohol as part of traditional cultural practices. Thus, educating the community on appropriate consumption of meat, targeting male members of the community is expected to have a substantial impact on the prevalence of taeniasis, although altering cultural dietary practices is recognized as a challenging approach to successful intervention. The most notable limitation of this study was the low prevalence of infection with individual species of *Taenia*, namely, *T. solium*, *T. saginata* and *T. asiatica* in study communities. This deterred our ability to build a model to predict risk factors associated with species-level *Taenia* infection in this study.

A three-stage cluster sampling design was used for this study with villages within districts as the primary sampling unit, households within villages as the secondary sampling unit and individuals within households as the tertiary sampling unit. Given the hierarchical structure of our data it was our a priori belief that observations made on individual study participants were not likely to be independent, justifying the use of regression modelling approaches that accounted for this non-independence. In our mixed-effects logistic regression model the variance of the random effect terms at both the village and individual household levels were negligible. Our inference is that in this population most of the unmeasured *Taenia*/cysticerci-exposure risks resided at the individual (as opposed to either the village or household) level. The practical implications of this finding are that in addition to addressing the modifiable risk factors identified in these analyses, health education programs need to be focused at the individual level.

Since many of the identified risk factors are traditional/behavioural practices in this area of Vietnam, the probability to induce permanent behavioural change from a single education campaign is likely to be small. A One Health approach utilising the expertise of anthropologists might be one way of effecting permanent behavioural change. Long term behavioural change in a population and on-going political support for health interventions occur when aspects of health risk, the economic benefits of disease control and the economic costs associated with disease can be quantified [40]. Financial and human resources are critical factors influencing the success of disease intervention programs. If resources are not available to support an integrated, long-term control and eradication program, a selective approach provides a suitable alternative with the selection of those interventions that provide the most cost-effective return on investment being preferred [41, 42]. The resource for taeniasis, cysticercosis intervention programs in Dak Lak province is limited; therefore, a selective approach is necessary. As discussed above, health education programs involving both the human medical and veterinary health sectors are a feasible intervention option. Choosing a small number of risk factors that make the greatest contribution to the prevalence of taeniasis or cysticercosis are logical targets within an education campaign, for example, indoor latrine construction will ultimately prove the most cost-effective approach.

## Conclusions

The prevalence of seroexposure to *T. solium* in the communities of Dak Lak is consistent with that of other regions in Vietnam. Identified risk factors are modifiable, and associated strongly with the prevalence of *T. solium* seroexposure and *Taenia* spp. infection in Dak Lak communities, thus advocating for targeted community intervention programs in mitigating these risks.

## Additional file

Additional file 1: Questionnaire 1 A blank copy of the questionnaire used in this study. (DOCX 178 kb)

## Abbreviations

CI: Confidence interval
LLGP-EITB: Lentil lectin purified glycoprotein enzyme-linked immunoelectrotransfer blot assay
OR: Odds ratio
T3qPCR: Multiplex real-time PCR
