# Supplementary material for: The epidemiology of Taenia spp. infection and Taenia solium cysticerci exposure in humans in the Central Highlands of Vietnam
Source: BMC Infect Dis. 2018 Oct 22;18:527. doi: 10.1186/s12879-018-3434-9 (PMC6198533; doi:10.1186/s12879-018-3434-9)
Supplement: Supplementary file 1 — Questionnaire 1 A blank copy of the questionnaire used in this study. (DOCX 178 kb) [file 12879_2018_3434_MOESM1_ESM.docx]

**QUESTIONNAIRE 1: GENERAL INFORMATION**

(PHẦN 1: THÔNG TIN CHUNG)

**CODE OF QUESTIONNAIR** *(mã câu hỏi):*

**Name of Interviewer** (tên người phỏng vấn):

**Date** (ngày):

***District*** *(huyện):*

1. M’DraK 2. Buon Don 3. KrongNang

***Commune*** *(Xã):*

*1. Krong Jing 4. Dlieya 7. Ea Nuôi*

*2. Cư M’ta 5. Ea Ver 8. Ea Tam*

*3. Ea Hồ 6. Krong Na 9. Ea Ba*

***Village:***

*(thôn)*

***House #:***

*(Số nhà)*

**Head of household (***Chủ hộ)***:**

**Number of people permanently living in household:**

*(Số người thường xuyên sống trong nhà)*

|  | ***1.Number***  *(Số người)* | ***2.Relationship with head of household***  *(quan hệ với chủ hộ)* |
| --- | --- | --- |
| 1. *0 – 7 years old (0-6 tuổi)* |  |  |
| 1. *7 – 17 years old (7 – 17 tuổi)* |  |  |
| 1. *18 – 30 years old (18 – 30 tuổi)* |  |  |
| 1. *31- 55 years old (31-55 tuổi)* |  |  |
| 1. *56 – 65 years old (56 – 65 tuổi)* |  |  |
| 1. *Oder than 65 years old (hơn 65 tuổi)* |  |  |

***🟅🟅🟅🟅🟅🟅***

1. **Where is drinking water sourced from?** (nguồn nước uống lấy từ đâu?)
2. *Wells (giếng)*
3. *Rain water (nước mưa)*
4. *Piped water (nước máy)*
5. *Stream/lake/pond (suối/hồ/ao)*
6. *Other (khác) ______________________*
7. **Which of the following methods do you treat water before drinking?** (phương pháp nào dùng để xử lý nước trước khi uống?)
8. *None (Không xử lý)*
9. *Boil (Nấu sôi)*
10. *Filter (Lọc)*
11. *Other (Khác) ______________________*
12. **Have you ever used manure to fertilize vegetables/crops?** (anh/chị có bao giờ sử dụng phân bón cho rau/cây trồng không?)
13. *Yes (có)*
14. *No (Không) go to question 6 (trả lời câu 6)*
15. **What kinds of manure are used and are they composted before use?**

| *Kind of fertilizer*  *(Loại phân sử dụng)* | *1. Yes*  *(Có)* | *2. No*  *(Không)* | *Composted*  *(ủ trước khi sử dụng)* | |
| --- | --- | --- | --- | --- |
|  |  |  | *1. Yes*  *(Có)* | *2. No*  *(Không)* |
| 1. *Human manure (phân người-phân bắc)* |  |  |  |  |
| 1. *Sheep manure (phân cừu)* |  |  |  |  |
| 1. *Cattle manure (phân bò)* |  |  |  |  |
| 1. *Buffalo manure(phân trâu)* |  |  |  |  |
| 1. *Goat manure (phân dê)* |  |  |  |  |
| 1. *Pig manure (phân heo)* |  |  |  |  |
| 1. *Chicken, duck manure (phân gia cầm )* |  |  |  |  |
| 1. *Chemical fertilizer* (phân hóa học)* |  |  |  |  |
| 1. *Microbial fertilizer^*^ (phân vi sinh)* |  |  |  |  |

*^*^Microbial fertilizer: E.g. NTK, Que Lam, Song Gianh, Viet Linh, Biogro*

*Chemical fertilizer: E.g. NPK, Dau Trau, Con O*

1. **How often do you fertilize your vegetables and/or crops?** (anh/chị có thường xuyên bón phân cho rau/cây trồng không?)
2. *Daily (hàng ngày)*
3. *Weekly (hàng tuần)*
4. *Monthly (hàng tháng)*
5. *Other (khác)______________________*
6. **What are the source(s) of water used for irrigating your vegetables/crops?** (nguồn nước sử dụng tưới rau/cây trông lấy từ đâu?)
7. *Wells (giếng)*
8. *Rain water (nước mưa)*
9. *Piped water (nước máy)*
10. *Stream/lake/pond (suối/hồ/ao)*
11. *Other (khác)______________________*
12. **Where is the stream/lake/pond located?** (vị trí của suối/hồ/ao ở đâu?)

|  | *1. Close to human defecation areas*  *(gần khu vệ sinh của người)* | *2. In garden/ back yard*  *(trong vườn/sau nhà)* | *3. Close to animal defecation areas*  *(gần chuồng vật nuôi)* | *4. Other*  *(nơi khác)* |
| --- | --- | --- | --- | --- |
| *1. Stream (suối)* |  |  |  |  |
| *2. Lake (hồ)* |  |  |  |  |
| *3. Pond (ao)* |  |  |  |  |
| *4. Giếng* |  |  |  |  |

1. **Does your house have a toilet/s?** (gia đình anh/chị có nhà vệ sinh không?)
2. *Yes (có)*
3. *No (không) go to question 11 (trả lời câu 11)*
4. **What kind of toilet do you have?** (nhà vệ sinh loại gì?)
5. *Septic tank (tự hoại)*
6. *Pit latrine (hố đào)*
7. *Composting latrine (hầm lấy phân)*
8. *Over hung latrine (cầu tõm)*
9. *Other(khác) ______________________*
10. **Where is the toilet located?** (vị trí của nhà vệ sinh?)
11. *Inside the main house (khép kín trong nhà)*
12. *In garden/backyard (vườn/sau nhà)*
13. *Over(near) stream/lake/pond (trên (gần) suối/hồ/ao)*
14. *Other (khác) ______________________*
15. **Your house information** *(thông tin về nhà cửa)*

| *Year of building*  *(năm xây, cất)* | *Material*  *(vật liệu xây dựng)* | *# of bedroom*  *(số phòng ngủ)* | *Floor information*  *(nền, sàn)* | | *Material of roof*  *(Vật liệu*  *mái nhà)* |
| --- | --- | --- | --- | --- | --- |
|  |  |  | *Material*  *(vật liệu)* | *Type*  *(kiểu)* |  |
|  | *1.* *Brick*  *2. Wood*  *3. Clay*  *4. Other* |  | *1. Granite*  *2. Cement*  *3. Ground*  *4. Wood*  *5. Other* | *1. On ground*  *2. Pile floor*  *3. Other* | *1. Brick*  *2. Wood*  *3. Clay*  *4. Other* |

1. **Stretch out a map showing location of house, garden, vegetable patch, pigsty and toilet** (vẽ sơ đồ, vị trí của nhà chính, vườn, khu trồng rau, chuồng heo và nhà vệ sinh)

**North**

1. **What species of domestic animals do you own?** (anh/chị hiện đang nuôi con gì?)

|  | Yes  (có nuôi) | Number  (số lượng) | No  (không nuôi) |
| --- | --- | --- | --- |
| 1. *Chicken-ducks (gà – vịt)* |  |  |  |
| 1. *Sheep (cừu)* |  |  |  |
| 1. *Cattle (bò)* |  |  |  |
| 1. *Buffalo (trâu)* |  |  |  |
| 1. *Goats (dê)* |  |  |  |
| 1. *Cats (mèo)* |  |  |  |
| 1. *Dogs (chó)* |  |  |  |
| 1. *Pigs (heo)* |  |  |  |

*If yes for pig or dog, please fill out section 3*

*(nếu có nuôi heo hay chó, làm ơn trả lời phần 3)*

**QUESTIONNAIRE 2: INDIVIDUAL INFORMATION**

(PHẦN 2: THÔNG TIN CÁ NHÂN)

**Name of the first person**:

(tên người thư nhất)

**Name of the second person:**

(tên người thứ 2)

**Name of the third person:**

(tên người thứ 3)

1^st^ 2^nd^ 3^rd^

(người 1) (người 2) (người 3)

- - - 1. **Age** (tuổi)

|  |  |  |
| --- | --- | --- |

- - - 1. **Gender** (giới tính)

| 1. *Male (nam)* |  |  |  |
| --- | --- | --- | --- |
| 1. *Female (nữ)* |  |  |  |

- - - 1. **Occupation** (nghề nghiệp)

| 1. *Office staff (nhân viên văn phòng)* | |  |  |  |
| --- | --- | --- | --- | --- |
| 1. *Business (kinh doanh, buôn bán)* | |  |  |  |
| 1. *Worker (công nhân)* | |  |  |  |
| *Farmer* | 1. *Rice grower (trồng lúa)* |  |  |  |
|  | 1. *Coffee grower (trồng cà phê)* |  |  |  |
|  | 1. *Pepper grower (trồng tiêu)* |  |  |  |
|  | 1. *Rubber grower (trồng cao su)* |  |  |  |
|  | 1. *Fisherman (ngư dân)* |  |  |  |
|  | 1. *Livestock rearer* *(chăn nuôi)* |  |  |  |
|  | 1. *Other (khác)* |  |  |  |

- - - 1. **Education** (trình độ học vấn)

| 1. *None (không học)* |  |  |  |
| --- | --- | --- | --- |
| 1. *Primary (tiểu học)* |  |  |  |
| 1. *Secondary (cấp hai)* |  |  |  |
| 1. *High school (cấp 3)* |  |  |  |
| 1. *University or higher (đại học)* |  |  |  |

- - - 1. **To which ethnic group do you belong?** (thành phần dân tộc?)

| 1. *Ede* |  |  |  |
| --- | --- | --- | --- |
| 1. *Kinh* |  |  |  |
| 1. *M’nong* |  |  |  |
| 1. *Thai* |  |  |  |
| 1. *Other* |  |  |  |

- - - 1. **To which religion do you belong?** (thành phần tôn giáo?)

| 1. *Free (không theo)* |  |  |  |
| --- | --- | --- | --- |
| 1. *Buddhist (đạo phật)* |  |  |  |
| 1. *Catholic* |  |  |  |
| 1. *Christian* |  |  |  |
| 1. *Other (khác)* |  |  |  |

- - - 1. **How often do you eat raw vegetables?** (anh/chị có thường ăn rau sống không?)

| - - - 1. *Daily (hàng ngày)* |  |  |  |
| --- | --- | --- | --- |
| - - - 1. *Several times/ week* *(vài lần/tuần)* |  |  |  |
| - - - 1. *Once/week (**một lần/tuần)* |  |  |  |
| - - - 1. *Once every two weeks* *(hai tuần/lần)* |  |  |  |
| - - - 1. *Less than one/ month* *(hiếm khi)* |  |  |  |
| - - - 1. *Never* *(không bao giờ)* |  |  |  |

- - - 1. **How often do you eat undercook dishes from pork?** (anh/chị có thường ăn món tái từ thịt heo không?)

| *1. Several times/ week* *(vài lần/tuần)* |  |  |  |
| --- | --- | --- | --- |
| *2. Once/week* *(một lần/tuần)* |  |  |  |
| *3. Once every two weeks* *(hai tuần/lần)* |  |  |  |
| *4. Less than one/month* *(hiếm khi)* |  |  |  |
| *5. Never* *(không bao giờ)* |  |  |  |

- - - 1. **How often do you eat “Nem”?** (anh/chị có thường ăn món “nem chua” không?)

| *1. Several times/ week (vài lần/tuần)* |  |  |  |
| --- | --- | --- | --- |
| *2. Once/week (một lần/tuần)* |  |  |  |
| *3. Once every two weeks (hai tuần/lần)* |  |  |  |
| *4. Less than one/ month* *(hiếm khi)* |  |  |  |
| *5. Never (không bao giờ)* |  |  |  |

- - - 1. **How often do you eat following organs of pig?** (anh/chị thường ăn những bộ phận nào từ heo?)

| *1. Liver*  *(gan heo)* | *1. Several times/ week (vài lần/tuần)* |  |  |  |
| --- | --- | --- | --- | --- |
|  | *2. Once/week (một lần/tuần)* |  |  |  |
|  | *3. Once every two weeks (hai lần/tuần)* |  |  |  |
|  | *4. Less than one/ month (hiếm khi)* |  |  |  |
|  | *5. Never (chưa bao giờ)* |  |  |  |
| *2. Tongue*  *(lưỡi heo)* | *1. Several times/ week (vài lần/tuần)* |  |  |  |
|  | *2. Once/week (một lần/tuần)* |  |  |  |
|  | *3. Once every two weeks (hai lần/tuần)* |  |  |  |
|  | *4. Less than one/ month (hiếm khi)* |  |  |  |
|  | *5. Never (chưa bao giờ)* |  |  |  |
| *3. Lean meat*  *(thịt nạc)* | *1. Several times/ week (vài lần/tuần)* |  |  |  |
|  | *2. Once/week (một lần/tuần)* |  |  |  |
|  | *3. Once every two weeks (hai lần/tuần)* |  |  |  |
|  | *4. Less than one/ month (hiếm khi)* |  |  |  |
|  | *5. Never (chưa bao giờ)* |  |  |  |
| *4. Brain*  *(óc heo)* | *1. Several times/ week (vài lần/tuần)* |  |  |  |
|  | *2. Once/week (một lần/tuần)* |  |  |  |
|  | *3. Once every two weeks (hai lần/tuần)* |  |  |  |
|  | *4. Less than one/ month (hiếm khi)* |  |  |  |
|  | *5. Never (chưa bao giờ)* |  |  |  |
| *5. Cheek*  *(thịt má)* | *1. Several times/ week (vài lần/tuần)* |  |  |  |
|  | *2. Once/week (một lần/tuần)* |  |  |  |
|  | *3. Once every two weeks (hai lần/tuần)* |  |  |  |
|  | *4. Less than one/ month (hiếm khi)* |  |  |  |
|  | *5. Never (chưa bao giờ)* |  |  |  |
| *6. Diaphgram*  *(cơ hoành)* | *1. Several times/ week (vài lần/tuần)* |  |  |  |
|  | *2. Once/week (một lần/tuần)* |  |  |  |
|  | *3. Once every two weeks (hai lần/tuần)* |  |  |  |
|  | *4. Less than one/ month (hiếm khi)* |  |  |  |
|  | *5. Never (chưa bao giờ)* |  |  |  |
| *7. Lung*  *(phổi heo)* | *1. Several times/ week (vài lần/tuần)* |  |  |  |
|  | *2. Once/week (một lần/tuần)* |  |  |  |
|  | *3. Once every two weeks (hai lần/tuần)* |  |  |  |
|  | *4. Less than one/ month (hiếm khi)* |  |  |  |
|  | *5. Never (chưa bao giờ)* |  |  |  |
| *8. Tim* | *1. Several times/ week (vài lần/tuần)* |  |  |  |
|  | *2. Once/week (một lần/tuần)* |  |  |  |
|  | *3. Once every two weeks (hai lần/tuần)* |  |  |  |
|  | *4. Less than one/ month (hiếm khi)* |  |  |  |
|  | *5. Never (chưa bao giờ)* |  |  |  |

- - - 1. **How often do you eat wild boar and/or free roaming pigs?** (anh/chị có thường ăn thịt heo rừng/heo đồng bào không?)

| *1. Several times per month (vài lần/tháng)* |  |  |  |
| --- | --- | --- | --- |
| *2. Once per month (tháng/lần)* |  |  |  |
| *3.* *Once every three months (ba tháng/lần)* |  |  |  |
| *4.* *Less than one per three months (ít hơn ba tháng/lần)* |  |  |  |
| *5. Never (chưa bao giờ)* |  |  |  |

- - - 1. **On which occasions do you consume these dishes (board/wild pig)?** (anh/chị ăn những món làm từ heo rừng/heo thả rông vào dịp nào?)

| 1. *Celebration events (lễ, hội)* |  |  |  |
| --- | --- | --- | --- |
| 1. *Wedding events (cưới hỏi)* |  |  |  |
| 1. *Funeral events (ma chay)* |  |  |  |
| 1. *Family meals (bữa ăn của gđình)* |  |  |  |
| 1. *Other (khác)* |  |  |  |

- - - 1. **How often have you ever seen white cysts (the size of rice grains) on the surface of pork selling at the market?** (anh/chị có thường thấy các hạt kén trên bề mặt thịt heo bán ở chợ không?)

| 1. *Often* *(thường xuyên)* |  |  |  |
| --- | --- | --- | --- |
| 1. *Sometime* *(thỉnh thoảng)* |  |  |  |
| 1. *Rarely* *(hiếm khi)* |  |  |  |
| 1. *Never* *(chưa bao giờ)* |  |  |  |

- - - 1. **How often have you ever seen white cysts (the size of rice grains) on the surface of pork (when you are cooking)?** (anh/chị có thường thấy các hạt kén trên bề mặt thịt heo khi nấu ăn không?)

| - 1. *Often (thường xuyên)* |  |  |  |
| --- | --- | --- | --- |
| - 1. *Sometime (thỉnh thoảng)* |  |  |  |
| - 1. *Rarely (hiếm khi)* |  |  |  |
| - 1. *Never (chưa bao giờ)* |  |  |  |

- - - 1. **Which part of the body are these cysts commonly lacated?** (những hạt kén đó thường thấy ở đâu trên thịt lợn?)

| 1. *Tongue (lưỡi heo)* |  |  |  |
| --- | --- | --- | --- |
| 1. *Diaphragm (cơ hoành)* |  |  |  |
| 1. *Liver (gan heo)* |  |  |  |
| 1. *Mesentery (màng treo ruột)* |  |  |  |
| 1. *Lean meat (thịt nạc)* |  |  |  |
| 1. *Brain (óc heo)* |  |  |  |
| 1. *Cheek (thịt má-tai)* |  |  |  |

- - - 1. **What do you do with this kind of pork?** (anh/chị đã xử lý như thế nào với những loại thịt đó?)

| 1. *Consume as usual (vẫn ăn)* |  |  |  |
| --- | --- | --- | --- |
| 1. *Don’t purchase (không mua)* |  |  |  |
| 1. *Discard (bỏ đi)* |  |  |  |
| 1. *Cook well and consume (nấu kỹ để sử dụng)* |  |  |  |

- - - 1. **How often do you eat beef?** (anh/chị có thường ăn thịt bò không?)

| *1. Several times/ week (vài lần/tuần)* |  |  |  |
| --- | --- | --- | --- |
| *2. Once/week (một lần/tuần)* |  |  |  |
| *3. Once every two weeks (hai tuần/lần)* |  |  |  |
| *4. Less than one/month (hiếm khi)* |  |  |  |
| *5. Never (chưa bao giờ)* |  |  |  |

- - - 1. **How often do you eat undercooked beef (e.g. beefsteak)?** (anh/chị có thường ăn món bò tái không?)

| *1. Several times/ week (vài lần/tuần)* |  |  |  |
| --- | --- | --- | --- |
| *2. Once/week (một lần/tuần)* |  |  |  |
| *3. Once every two weeks (hai tuần/lần)* |  |  |  |
| *4. Less than one/month (hiếm khi)* |  |  |  |
| *5. Never (chưa bao giờ)* |  |  |  |

- - - 1. **Do you have any children under 7 years of age?** (anh/chị có con nhỏ dưới 7 tuổi không?)

| 1. *Yes (có)* |  |  |  |
| --- | --- | --- | --- |
| 1. *No (không)* |  |  |  |

***If “No”, please go to question 22*** *(nếu “không”, trả lời câu 22)*

- - - 1. **Where do they defecate?** (con anh/chị đi vệ sinh ở đâu?)

| 1. *Toilet (nhà vệ sinh)* |  |  |  |
| --- | --- | --- | --- |
| 1. *Pigsty (chuồng heo)* |  |  |  |
| 1. *Lake/pond/stream (hồ/ao/suối)* |  |  |  |
| 1. *Back yard/Garden (sau nhà/vườn)* |  |  |  |
| 1. *Potty (bô)* |  |  |  |
| 1. *Other (khác)* |  |  |  |

***If “5” is chosen,*** ***please go to question 21*** *(nếu chọn “5”, trả lời câu 21)*

- - - 1. **If they use potty, what do you do with their stool? Dispose to** (nếu con anh/chị đi vệ sinh vào bô, phân của chúng đổ ở đâu?)

| 1. *Toilet (nhà vệ sinh)* |  |  |  |
| --- | --- | --- | --- |
| 1. *Pigsty (chuồng heo)* |  |  |  |
| 1. *Lake/pond/stream (hồ/ao/suối)* |  |  |  |
| 1. *Back yard/garden (sau nhà/vườn)* |  |  |  |
| 1. *Other (khác)* |  |  |  |

- - - 1. **Where do you defecate**? (anh/chị đi vệ sinh ở đâu)

| 1. *Toilet (nhà vệ sinh)* |  |  |  |
| --- | --- | --- | --- |
| 1. *Pigsty (chuồng heo)* |  |  |  |
| 1. *Lake/pond/stream (hồ/ao/suối)* |  |  |  |
| 1. *Back yard/garden (sau nhà/vườn)* |  |  |  |
| 1. *Other (khác)* |  |  |  |

- - - 1. **Have you ever seen white** **proglottids in stool?** (anh/chị có thường thấy đốt sán trong phân không?)

| 1. *Yes (có)* |  |  |  |
| --- | --- | --- | --- |
| 1. *No (không)* |  |  |  |

***If “No”, please go to question 26*** *(nếu “không”, trả lời câu 26)*

- - - 1. **To whom did the stool with proglottids belong?** (phân có đốt sán thuộc về ai?)

| 1. *Yours (của anh/chị)* |  |  |  |
| --- | --- | --- | --- |
| 1. *Member in family (người gia đình)* |  |  |  |
| 1. *Neighbors’ (hàng xóm)* |  |  |  |

- - - 1. **How were the stool treated?** (loại phân đó đã được xử lý như thế nào?)

| 1. *Treated as usual (như thường lệ)* |  |  |  |
| --- | --- | --- | --- |
| 1. *Buried (chôn)* |  |  |  |
| 1. *Burnt (đốt)* |  |  |  |
| 1. *Other (khác)* |  |  |  |

- - - 1. **Do you wash your hands after defecating?** (anh/chị có rửa tay sau khi đi vệ sinh không?)

| 1. *Yes (có)* |  |  |  |
| --- | --- | --- | --- |
| 1. *No (không)* |  |  |  |
| 1. *Sometimes (thỉnh thoảng)* |  |  |  |

- - - 1. **Do your children wash their hands after defecating?** (con anh/chị có rửa tay sau kkhi đi vệ sinh không?)

| 1. *Yes (có)* |  |  |  |
| --- | --- | --- | --- |
| 1. *No (không)* |  |  |  |
| 1. *Sometimes (thỉnh thoảng)* |  |  |  |

- - - 1. **Do you wash your hands before eating?** (anh/chị có rửa tay trước khi ăn không?)

| 1. *Yes (có)* |  |  |  |
| --- | --- | --- | --- |
| 1. *No (không)* |  |  |  |
| 1. *Sometimes (thỉnh thoảng)* |  |  |  |

- - - 1. **Do your children wash their hands before eating?** (con anh/chị có rửa tay trước khi ăn không?)

| 1. *Yes (có)* |  |  |  |
| --- | --- | --- | --- |
| 1. *No (không)* |  |  |  |
| 1. *Sometimes (thỉnh thoảng)* |  |  |  |

- - - 1. **Do you wear footwear at all times when outdoor?** (anh/chị có mang dép khi đi ra ngoài không?)

| 1. *Yes (có)* |  |  |  |
| --- | --- | --- | --- |
| 1. *No (không)* |  |  |  |
| 1. *Sometimes (thỉnh thoảng)* |  |  |  |

- - - 1. **How often do you get weakness?** (anh chị có hay cảm thấy mệt mỏi không?)

| 1. *Often (thường xuyên)* |  |  |  |
| --- | --- | --- | --- |
| 1. *Sometime (thỉnh thoảng)* |  |  |  |
| 1. *Rarely (hiếm khi)* |  |  |  |
| 1. *Never (chưa bao giờ)* |  |  |  |

- - - 1. **Chek muscous membrance for pallor** (trạng thái niêm mạc miệng, mắt, lưỡi của anh/chị)

| 1. *Pallor (nhợt nhạt)* |  |  |  |
| --- | --- | --- | --- |
| 1. *Normal (bình thường)* |  |  |  |

- - - 1. **How often do you have epilepsy?** (anh/chị có hay bị chứng động kinh không?)

| *1. Often (thường xuyên)* |  |  |  |
| --- | --- | --- | --- |
| *2. Sometime (thỉnh thoảng)* |  |  |  |
| *3. Rarely (hiếm khi)* |  |  |  |
| *4. Never (chưa bao giờ)* |  |  |  |

- - - 1. **How often do you get headache?** (anh/chị có hay bị đau đầu không?)

| *1. Often (thường xuyên)* |  |  |  |
| --- | --- | --- | --- |
| *2. Sometime (thỉnh thoảng)* |  |  |  |
| *3. Rarely (hiếm khi)* |  |  |  |
| *4. Never (chưa bao giờ)* |  |  |  |

- - - 1. **How often do you have nausea/vomiting** (anh/chị có hay buồn nôn hay nôn không?)

| *1. Often (thường xuyên)* |  |  |  |
| --- | --- | --- | --- |
| *2. Sometime (thỉnh thoảng)* |  |  |  |
| *3. Rarely (hiếm khi)* |  |  |  |
| *4. Never (chưa bao giờ)* |  |  |  |

- - - 1. **How often do you have numbness/achy limbs** (anh/chị có hay bị tê/đau nhức chân tay không?)

| *1. Often (thường xuyên)* |  |  |  |
| --- | --- | --- | --- |
| *2. Sometime (thỉnh thoảng)* |  |  |  |
| *3. Rarely (hiếm khi)* |  |  |  |
| *4. Never (chưa bao giờ)* |  |  |  |

- - - 1. **How often do you suffer from temporary paralysis?** (anh/chị có thường không cử động chân tay được trong thời gian ngắn không?)

| *1. Often (thường xuyên)* |  |  |  |
| --- | --- | --- | --- |
| *2. Sometime (thỉnh thoảng)* |  |  |  |
| *3. Rarely (hiếm khi)* |  |  |  |
| *4. Never (chưa bao giờ)* |  |  |  |

- - - 1. **How often do you have muscle pain?**(anh/chị có thường bị đau nhức cơ không?)

| *1. Often (thường xuyên)* |  |  |  |
| --- | --- | --- | --- |
| *2. Sometime (thỉnh thoảng)* |  |  |  |
| *3. Rarely (hiếm khi)* |  |  |  |
| *4. Never (chưa bao giờ)* |  |  |  |

- - - 1. **How often do you have fidgeted anus?** (anh/chị có thường cảm thấy ngứa ngáy, nhột nhạt ở hậu môn không?)

| *1. Often (thường xuyên)* |  |  |  |
| --- | --- | --- | --- |
| *2. Sometime (thỉnh thoảng)* |  |  |  |
| *3. Rarely (hiếm khi)* |  |  |  |
| *4. Never (chưa bao giờ)* |  |  |  |

- - - 1. **How often do you have abdominal pain?** (anh/chị có thường bị đau bụng không?)

| *1. Often (thường xuyên)* |  |  |  |
| --- | --- | --- | --- |
| *2. Sometime (thỉnh thoảng)* |  |  |  |
| *3. Rarely (hiếm khi)* |  |  |  |
| *4. Never (chưa bao giờ)* |  |  |  |

- - - 1. **How often do you have diarrhoea?** (anh/chị có thường bị tiêu chảy không?)

| *1. Often (thường xuyên)* |  |  |  |
| --- | --- | --- | --- |
| *2. Sometime (thỉnh thoảng)* |  |  |  |
| *3. Rarely (hiếm khi)* |  |  |  |
| *4. Never (chưa bao giờ)* |  |  |  |

- - - 1. **Do you have any cysts (nodules) under your skin?** (dưới da của anh/chị có xuất hiện các u, cục không?)

| 1. *Yes (có)* |  |  |  |
| --- | --- | --- | --- |
| 1. *No (không)* |  |  |  |

***If” No” is chosen, please go to question 44*** *(nếu chọn “không”, trả lời câu hỏi 44)*

- - - 1. **Please describe these nodules?** (anh/chị làm ơn mô tả các u, cục?)

| *1. Location (vị trí)* |  |  |  |
| --- | --- | --- | --- |
| *2. Size (kích thước)* |  |  |  |
| *3. Morphology (hình dáng)* |  |  |  |
| *4. Duration of exist (thời gian tồn tại)* |  |  |  |

- - - 1. **Have you ever heard about** **taeniasis/****porcine cysticercosis/****trichinellosis?** (anh/chị có bao giờ nghe về bệnh sán xơ mít, gạo lợn, giun bao không?)

| *1. Taeniasis*  *(sán xơ mít)* | *1. Yes (có)* |  |  |  |
| --- | --- | --- | --- | --- |
|  | *2. No (không)* |  |  |  |
| *2. Porcine cysticercosis*  *(gạo lợn)* | *1. Yes (có)* |  |  |  |
|  | *2. No (không)* |  |  |  |
| *3. Trichinellosis*  *(giun bao)* | *1. Yes (có)* |  |  |  |
|  | *2. No (không)* |  |  |  |

- - - 1. **What do you know about taeniasis?** (anh/chị hiểu gì về bệnh sán xơ mít)

| ***Grade 1*** *(Knew about the cause, effect of taeniasis, how to avoid and prevent)*  *(****mức 1:*** *biết* *nguyên nhân, hậu quả, cách phòng và trị)* |  |  |  |
| --- | --- | --- | --- |
| ***Grade 2*** *(Knew Vaguely about the cause, effect of taeniasis, how to avoid and prevent)*  *(****mức 2:*** *biết một cách mơ hồ về nguyên nhân, hậu quả, cách phòng và trị)* |  |  |  |
| ***Grade 3*** *(Do not know completely)*  *(****mức 3:*** *hoàn toàn không biết)* |  |  |  |

- - - 1. **What do you know about** **porcine cysticercosis?** (anh/chị biết gì về gạo lợn?)

| ***Grade 1*** *(Knew about the cause, effect of taeniasis, how to avoid and prevent)*  *(****mức 1:*** *biết nguyên nhân, hậu quả, cách phòng và trị)* |  |  |  |
| --- | --- | --- | --- |
| ***Grade 2*** *(Knew Vaguely about the cause, effect of taeniasis, how to avoid and prevent)*  *(****mức 2:*** *biết một cách mơ hồ về nguyên nhân, hậu quả, cách phòng và trị)* |  |  |  |
| ***Grade 3*** *(Do not know completely)*  *(****mức 3:*** *hoàn toàn không biết)* |  |  |  |

- - - 1. **What do you know about trichinellosis?** (anh/chị biết gì về bệnh giun bao?)

| ***Grade 1*** *(Knew about the cause, effect of taeniasis, how to avoid and prevent)*  *(****mức 1:*** *biết nguyên nhân, hậu quả, cách phòng và trị )* |  |  |  |
| --- | --- | --- | --- |
| ***Grade 2*** *(Knew Vaguely about the cause, effect of taeniasis, how to avoid and prevent)*  *(****mức 2:*** *biết một cách mơ hồ về nguyên nhân, hậu quả, cách phòng và trị)* |  |  |  |
| ***Grade 3*** *(Do not know completely)*  *(****mức 3:*** *hoàn toàn không biết)* |  |  |  |

- - - 1. **Where do you get the information?** (anh/chị biết những thông tin trên ở đâu?)

| *1.Periodical (sách, báo, tạp chí)* |  |  |  |
| --- | --- | --- | --- |
| *2.Radio/TV/the Internet (đài/tivi/internet)* |  |  |  |
| *3.Medical officers (nhân viên y tế)* |  |  |  |
| *4.Other (khác)* |  |  |  |

- - - 1. **Are there any members in your family have been diagnosed infected with taeniasis/cysticercosis/trichinellosis?** (gia đình anh/chị có ai bị các bệnh trên không?)

| *1. Taeniasis*  *(sán xơ mít)* | *1. Yes (có)* |  |  |  |
| --- | --- | --- | --- | --- |
|  | *2. No (không)* |  |  |  |
| *2. Porcine cysticercosis*  *(gạo lợn)* | *1. Yes (có)* |  |  |  |
|  | *2. No (không)* |  |  |  |
| *3. Trichinellosis*  *(giun bao)* | *1. Yes (có)* |  |  |  |
|  | *2. No (không)* |  |  |  |

- - - 1. **Are they treated?** (họ đã được chữa khỏi chưa?)

| *1. Taeniasis*  *(sán xơ mít)* | *1. Yes (có)* |  |  |  |
| --- | --- | --- | --- | --- |
|  | *2. No (không)* |  |  |  |
| *2. Porcine cysticercosis*  *(gạo lợn)* | *1. Yes (có)* |  |  |  |
|  | *2. No (không)* |  |  |  |
| *3. Trichinellosis*  *(giun bao)* | *1. Yes (có)* |  |  |  |
|  | *2. No (không)* |  |  |  |

- - - 1. **What have you done to protect you and your family from parasitic diseases?** (anh/chị làm gì để bảo vệ gia đình khỏi các bệnh do giun sán?)

| ***Grade 1*** *(deworm regularly, avoid eating undercook meat or raw vegetable,* *wash hand before eat and after defecation)*  *(****Mức 1:*** *tẩy giun định kỳ, tránh ăn món tái, sống, rửa tay trước khi ăn và sau khi đi vệ sinh)* |  |  |  |
| --- | --- | --- | --- |
| ***Grade 2*** *(sometime deworm, still eat* *undercook dishes, sometime* *wash hand before eat and after defecation)*  *(****Mức 2:*** *thỉnh thoảng tẩy giun, ăn món tái, sống, thỉnh thoảng rửa tay trước khi ăn và sau khi đi vệ sinh)* |  |  |  |
| ***Grade 3*** *(seldom deworm, eating undercook meat or raw vegetable, not aware of transmitting parasites via hand-mouth*  *(****Mức 3:*** *hiếm khi tẩy giun, ăn món ăn tái, sống, không ý thức được vệ lây nhiễm giun sán theo đường tay, chân – miệng)* |  |  |  |
| ***Grade 4*** *(Not aware of parasitic diseases completely and do nothing)*  *(****Mức 4:*** *hoàn toàn không ý thức được bệnh giun sán và không thực hiện bất cứ quy tắc vệ sinh nào)* |  |  |  |

- - - 1. **How often do you deworm for you and family members?** (anh/chị có thường xuyên tẩy giun sán cho bản than và thành viên gia đình không?)

| 1. *Never (không bao giờ)* |  |  |  |
| --- | --- | --- | --- |
| 1. *Once a year (năm/lần)* |  |  |  |
| 1. *Twice a year (hai lần/năm* |  |  |  |
| 1. *Every two years (hai năm 1 lần)* |  |  |  |
| 1. *Longer (lâu hơn)* |  |  |  |

- - - 1. **What medication do you use for deworming?** (anh/chị sử dụng loại thuốc nào để tẩy giun sán?)

| *1^st^ person (người thứ 1)* |  |
| --- | --- |
| *2^nd^ person (người thứ 2)* |  |
| *3^rd^ person (người thứ 3)* |  |

**QUESTIONNAIRE 3: PIGS/DOGS INFORMATION**

(PHẦN 3: THÔNG TIN VỀ HEO/CHÓ)

**CODE OF QUESTIONNAIR** (mã câu hỏi):

**Name of Interviewer** (người phỏng vấn):

**Date** (ngày):

***District*** *(huyện):*

1. M’DraK 2. Buon Don 3. KrongNang

***Commune*** *(xã):*

*1. CuM’ta 1. Ea Nuoi 1. Ea Tan*

*2. Krongjing 2. Tan Hoa 2. Phu Loc*

*3. Ea Rieng 3. Krong Na 3. Phu Xuan*

***Village:***

*(buôn/làng)*

***House #***

*(số nhà)*

**Head of household** (chủ hộ):

**Number of Pigs** (số lượng heo nuôi):

Pig A Pig B Pig C

(Heo A) (Heo B) (Heo C)

| 1. **Age (month)** (tháng tuổi) |  |  |  |
| --- | --- | --- | --- |
| 1. **Sex** (tính biệt) |  |  |  |
| 1. **Breed** (giống) |  |  |  |

1. **Are your pigs confined or been free roaming?** (heo của anh/chị được nuôi nhốt hay nuôi thả)

| 1. *Confined (nhốt)* |  |  |  |
| --- | --- | --- | --- |
| 1. *Free roaming (thả)* |  |  |  |
| 1. *Both (cả hai)* |  |  |  |

1. **Do you have a pigsty?** (nhà anh/chị có chuồng heo không?)

| 1. *Yes (có)* |  |  |  |
| --- | --- | --- | --- |
| 1. *No (không)* |  |  |  |

***If “No”, please go to question 7*** *(nếu “không”, trả lời câu 7)*

1. **Where is the pigsty located?** (vị trí của chồng heo ở đâu?)

| 1. *Close to the main house (gần nhà chính)* |  |  |  |
| --- | --- | --- | --- |
| 1. *In garden/backyard (trong vườn/sau nhà chính)* |  |  |  |
| 1. *Over(near) lake/stream/pond (trên/gần hồ/suối/ao)* |  |  |  |
| 1. *Other (khác)* |  |  |  |

1. **Do your pigs have access to areas where humans defecate?** (heo có bao giờ đi vào khu vực vệ sinh của người không?)

| 1. *Yes (có)* |  |  |  |
| --- | --- | --- | --- |
| 1. *No (không)* |  |  |  |

1. **How often do your pigs eat human stools?** (heo có thường xuyên ăn phân người không?)

| 1. *Daily (hàng ngày)* |  |  |  |
| --- | --- | --- | --- |
| 1. *Sometime (thỉnh thoảng)* |  |  |  |
| 1. *Never (không bao giờ)* |  |  |  |

1. **What kinds of food are your pigs fed?** (anh/chị nuôi heo bằng thức ăn gì?)

| 1. *Commercial food (cám công nghiệp)* |  |  |  |
| --- | --- | --- | --- |
| 1. *Human left-over raw food (đồ ăn sống của người)* |  |  |  |
| 1. *Human left-over cooked food (thức ăn chín thừa của người)* |  |  |  |
| 1. *Scavenge for themselvesn (tự kiếm)* |  |  |  |
| 1. *Other (khác)* |  |  |  |

1. **How often do your pigs eat animal cadavers (e.g. rats, other pigs)?** (anh/chị có thấy heo ăn xác chết của động vật khác không (chuột chết, heo chết)?)

| *1. Often (thường xuyên)* |  |  |  |
| --- | --- | --- | --- |
| *2. Sometime (thỉnh thoảng)* |  |  |  |
| *3. Rarely (hiếm khi)* |  |  |  |
| *4. Never (chưa bao giờ)* |  |  |  |

1. **How far do your pigs roam?** (heo của anh/chị thường đi rông bao xa?)

| 1. *Around forest border (đến bìa rừng)* |  |  |  |
| --- | --- | --- | --- |
| 1. *Around village (đi khắp làng)* |  |  |  |
| 1. *Around back yard (trong khu vực đất gia đình)* |  |  |  |
| 1. *Do not know (không biết)* |  |  |  |

1. **Where do your dogs defecate?** (chó của anh/chị đi vệ sinh ở đâu?)

| *1. Garden/ back yard (trong vườn/sau nhà)* |  |  |  |
| --- | --- | --- | --- |
| *2. Inside the house (trong nhà ở chính)* |  |  |  |
| *3. Outside house ground (ngoài khu đất gia đình)* |  |  |  |
| *4. Other (khác)* |  |  |  |

1. **What do you with dogs faeces? Dispose to** (anh/chị làm gì với phân có thải ra?) vứt vào:

| 1. *Do nothing (không làm gì)* |  |  |  |
| --- | --- | --- | --- |
| 1. *Toilet (nhà vệ sinh)* |  |  |  |
| 1. *Land around house (ngoài nhà)* |  |  |  |
| 1. *Stream/lake/pond (suối/hồ/ao)* |  |  |  |
| 1. *Pigsty (chuồng heo)* |  |  |  |
| 1. *Other (khác)* |  |  |  |

1. **How often have you seen proglottid in dog’s faeces?** (anh/chị có thường thấy đốt sán ở phân chó không?)

| *1. Often (thường xuyên)* |  |  |  |
| --- | --- | --- | --- |
| *2. Sometime (thỉnh thoảng)* |  |  |  |
| *3. Rarely (hiếm khi)* |  |  |  |
| *4. Never (chưa bao giờ)* |  |  |  |

1. **How often do you deworm for your dogs?** (anh/chị có thường tẩy giun sán cho chó không?)

| 1. *Never (chưa bao giờ)* |  |  |  |
| --- | --- | --- | --- |
| 1. *Every three months (3 tháng/lần)* |  |  |  |
| 1. *Every six months (6 tháng/lần)* |  |  |  |
| 1. *Other (khác)* |  |  |  |

1. **What medication do you use for your dogs?** (loại thuốc nào anh/chị dùng để tẩy giun cho chó?)

| *1^st^* *dog (chó 1)* |  |
| --- | --- |
| *2^nd^ dog (chó 2)* |  |
| *3^rd^ dog (chó 3)* |  |
